# New Therapeutic Horizon of Graves’ Hyperthyroidism: Treatment Regimens Based on Immunology and Ingredients From Traditional Chinese Medicine

**DOI:** 10.3389/fphar.2022.862831

**Published:** 2022-04-05

**Authors:** Qiongyao He, Hui Dong, Minmin Gong, Yujin Guo, Qingsong Xia, Jing Gong, Fuer Lu

**Affiliations:** ^1^ Institute of Integrated Traditional Chinese and Western Medicine, Tongji Hospital, Tongji Medical College, Huazhong University of Science and Technology, Wuhan, China; ^2^ Grade 2017 of Integrated Traditional Chinese and Western Clinical Medicine, Second Clinical School, Tongji Hospital, Tongji Medical College, Huazhong University of Science and Technology, Wuhan, China; ^3^ Department of Integrated Traditional Chinese and Western Medicine, Tongji Medical College, Tongji Hospital, Huazhong University of Science and Technology, Wuhan, China

**Keywords:** graves’ ophthalmopathy, graves’ disease (GD), traditional chinese medicine, immunology, treatment regimens

## Abstract

Graves’ disease is an autoimmune disease characterized by goiter and hyperthyroidism, and 25% patients develop GO. Traditional treatment options, such as antithyroid drugs, radioiodine or thyroidectomy, have remained largely unchanged over the past 70 years. For many patients, there is a high rate of recurrence after antithyroid drugs and lifelong hypothyroidism after ablation and thyroidectomy. The symptoms and quality of life of some patients have not been effectively improved. The clinical demand for new therapeutic regimens, coupled with a deeper understanding of the pathophysiology and immunobiology of Graves’ disease, has led to the emergence of several new therapeutic ideas, including biologics, small molecule peptides, immunomodulators and teprotumumab, a specific antibody targeting IGF-1R. Besides, the elements of TCM have attracted more and more interests in modern medicine, because some effective components have been successfully used in the treatment of autoimmune diseases. Based on the pathophysiology and efficacy of clinical management and treatment in Graves’ hyperthyroidism, here we review the new strategies under investigation and summarize the effective components of traditional Chinese medicine used for Graves’ hyperthyroidism, and explore their mechanisms. These therapies have opened a new window for the treatment of Graves’ disease, but the exact mechanism and the research direction still need to be further explored.

## Highlights

1) The pathogenesis of Graves’ disease is that thyroid stimulating hormone receptor (TSHR) antigen secreted by thyroid gland is specifically recognized by immune cells and reactive thyroid stimulating hormone receptor antibody (TRAB) is secreted to act on thyroid follicular cells and orbital fibroblasts_._
2) Antithyroid drugs, radioactive iodine and thyroidectomy are three traditional treatment methods. Now, biological agents, small molecule peptides, immunomodulators and antibodies targeting growth factor-I receptor (IGF-1R) have also been proposed_._
3) There are risks of disease recurrence, lifelong replacement therapy of thyroid hormone and complications associated with traditional treatment regimens, while other new treatment regimens have limitations_._
4) The effective components of traditional Chinese medicine can effectively relieve large goiter and reduce side effects of antithyroid drugs (ATD), relieve symptoms of Graves’ ophthalmopathy (GO), alleviate the hypermetabolic symptoms of Graves’ disease (GD), reduce allergic symptoms and increase the dosage of ATD used in allergic patients_._
5) The mechanism of effective components of traditional Chinese medicine (TCM) is different from the existing treatment projects, and it still needs a lot of high-quality randomized controlled trial (RCT) studiesand in-depth explorations to confirm the efficacy and elucidate the exact mechanism_._

## Introduction

GD is an organ-specific autoimmune disease that causes excessive thyroid hormone secretion (hyperthyroidism). GD is the most common cause of persistent hyperthyroidism in adults. Approximately 3% of women and 0.5% of men will develop Graves’ disease during their lifetime (Burch and Cooper, 2015). It is clinically characterized by thyrotoxicosis, serum anti-thyroid antibodies (ATA) and the presence of autoreactive lymphocytes in the glands. Thyroid stimulating hormone (TSH) receptor (TSHR), thyroid peroxidase (TPO) and thyroglobulin (TG) have unusual properties (“ immunogenicity”) and can disrupt immune tolerance. (Burch and Cooper, 2015). Abnormal release of thyroid hormone affects many body systems, so signs and symptoms associated with GD can vary widely and can significantly affect overall health. Common symptoms include shaking, heat sensitivity and warmth, loss of weight even with normal eating habits, anxiety and irritability, goiter, changes of menstrual cycle, erectile dysfunction and loss of libido, fatigue, frequent bowel movements, palpitations, etc. At present, ATD are the first-line treatment for GD. Ablative therapy with radioactive iodine (RAI) or surgical thyroidectomy can lead to hypothyroidism and lifelong replacement therapy of thyroid hormone. High-dose intravenous immunoglobulin or corticosteroid (CS) can reduce inflammation and orbital congestion in patients with active GO. Orbital decompression surgery and post-globular radiotherapy are also selectable treatment methods for Graves’ oculopathy, but side effects limit their widespread application.

The clinical demand for new therapeutic regimens of Graves’ disease has led to the emergence of several new therapeutic ideas, including biologics, small molecule peptides, immunomodulators and teprotumumab. Elements of TCM have also attracted more and more interests in modern medicine because they have unique curative effects and mechanisms of action. Nutraceuticals are included in TCM, which are defined as a food, or parts of a food, that provide medical or health benefits, including the prevention of different pathological conditions, and thyroid diseases, or the treatment of them. Nutraceuticals have a place in complementary medicines, being positioned in an area among food, food supplements, and pharmaceuticals. The market of certain nutraceuticals such as thyroid supplements has been growing in the last years (Benvenga et al., 2020). Based on the pathology and efficacy of clinical management and treatment in Graves’ hyperthyroidism, this article summarizes the new strategies under investigation and the effective components of traditional Chinese medicine used for Graves’ hyperthyroidism, and explores corresponding mechanisms.

## Pathophysiology

GD is an autoimmune disease caused by the abnormally activated immune system and the loss of immune tolerance to TSHR (Smith and Hegedüs, 2016). TSHR peptide secreted by thyroid tissue is taken up by dendritic cells, and the major histocompatibility complex (MHC) class II molecules on the surface of dendritic cells bind to T cell antigen receptor (TCR) on the surface of T lymphocytes to present TSHR peptide to T lymphocytes. Once CD154 molecules on the surface of activated T lymphocytes recognize CD40 molecules on the surface of B cells, the CD40-CD154 interaction initiates the costimulatory pathway, providing the first signal needed to initiate the adaptive humoral immune response (Smith and Hegedüs, 2016). This interaction between B and T lymphocytes is thought to play a central role in the pathogenesis of GD, as it is required for the formation and maturation of B cells in the germinal centers of the thyroid, permitting the production of pathogenic thyroid-stimulating antibodies (Armengol et al., 2001; Huber et al., 2012; Smith and Hegedüs, 2016). B cell activating factor (BAFF) is a member of the tumor necrosis factor (TNF) family of cytokines that plays an important role in the activation, differentiation and survival of B lymphocytes. Elevated circulating BAFF levels have been found in patients with various autoimmune diseases, including GD, in which elevated levels of thyroid hormone and TRAB have been shown to correlate with serum BAFF levels (Lin et al., 2016). The interaction of BAFF and BAFF receptor on the surface of B lymphocytes is the second signal to initiate adaptive humoral immune response.

**FIGURE 1:**
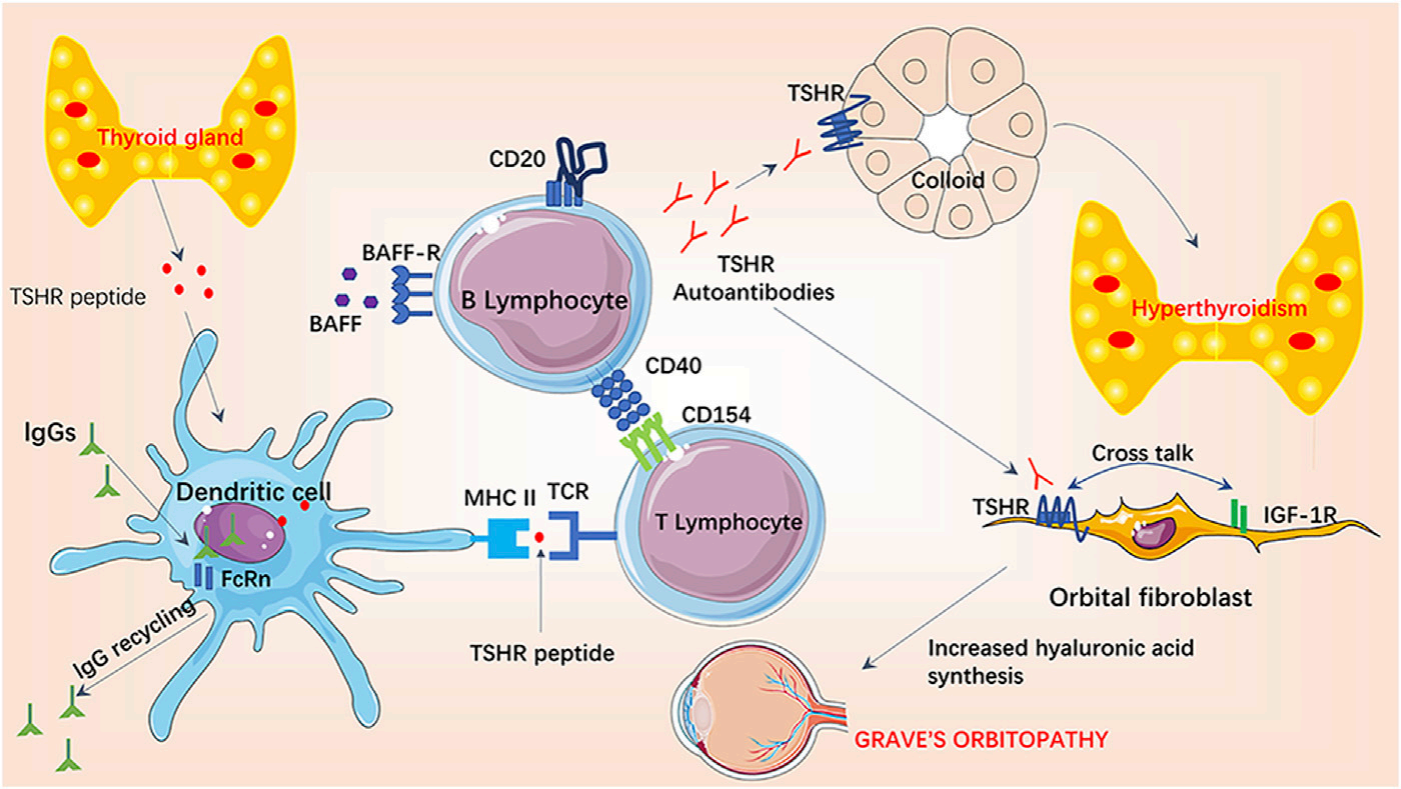
Pathogenesis of Graves’ hyperthyroidism and Graves’ orbitopathy.

After the humoral immune response is activated, B lymphocytes secrete large amounts of TRAB, which can be divided into two forms: stimulating type and blocking type (Diana et al., 2016). Circulatory stimulating TRAB is similar to TSH agonist and specifically binds to TSHR to stimulate the proliferation and hypertrophy of thyroid cell, promoting the expression of sodium-iodine cotransporter, thyroglobulin and thyroid peroxidase genes, and ultimately promote the production of thyroid hormone and hyperthyroidism (Smith and Hegedüs, 2016). Blocking TRAB has no functional activity in combination with TSHR and is considered as a neutralizing antibody. TRAB also acts on TSHR on the surface of orbital fibroblasts, leading to the release of hydrophilic mucopolysaccharide and pro-inflammatory cytokines, which intensifies the orbital inflammatory process, leading to local edema, congestion and eyeball herniation (Armengol et al., 2001; Graves' disease, 2000). Moreover, recent *in vitro* experiments have shown that insulin-like growth factor-1 receptor (IGF-1R) is another key player and associated autoantigen in the pathogenesis of GO (Wang and Smith, 2014). TSHR and IGF-1R co-locate to thyroid cells and orbital fibroblasts (Smith and Janssen, 2019). When TRAB binds to cells expressing TSHR ligands, “crosstalk” between TSHR and IGF-1R leads to activation of IGF-1R -dependent downstream intracellular pathways (Krieger et al., 2016; Krieger et al., 2017; Marcus-Samuels et al., 2018; Krieger et al., 2019). T helper 1 (Th1) immune response prevails in the immunopathogenesis of GD and GO, during which Th1 chemokines, and the chemokine receptor (C-X-C) R3, play a key role. In GD, recruited Th1 lymphocytes lead to an increased IFN-*γ* and TNF-α production, that stimulates Th1 chemokines secretion from thyroid cells, reiterating the autoimmune process. Elevated serum Th1 chemokines levels are associated with the active phases of GD (Antonelli et al., 2020). (Figure 1)

TSHR peptide secreted by thyroid tissue is taken up by dendritic cells, then MHC class II molecules on the surface of dendritic cells bind to TCR on the surface of T lymphocytes to present TSHR peptide to T lymphocytes. Once CD154 molecules on the surface of activated T lymphocytes recognizes CD40 molecules on the surface of B cells, the CD40-CD154 interaction initiates the costimulatory pathway, providing the first signal needed to initiate the adaptive humoral immune response, then the interaction of BAFF and BAFF receptor on the surface of B lymphocytes provides the second signal. After the humoral immune response is activated, B lymphocytes secrete large amounts of TRAB, of which stimulating type binds to TSHR. TSHR can co-locate with IGF-1R on thyroid cells and orbital fibroblasts to activate subsequent hyperthyroidism and GO.

TSHR, thyroid stimulating hormone (TSH) receptor; BAFF, B cell activating factor; IGF-1R, insulin-like growth factor-I receptor; FcRn, neonatal immunoglobulin receptor.

## Management of Graves’ Hyperthyroidism and Negative Effects

Currently, there are three traditional methods for the treatment of Graves’ hyperthyroidism: drugs to inhibit thyroid hormone production, thyroidectomy and thyroid tissue contraction induced by RAI. To date, ATD have been the preferred method for patients worldwide (Bartalena, 2013; Kahaly et al., 2018). ATD inhibits iodination, a process catalyzed by thyroid peroxidase, of which methimazole (MMI) is a classical and widely distributed ATD (Taurog et al., 1976; Davidson et al., 1978). By contrast, Carbimazole (CBM), an inactive drug, is in much smaller supply worldwide. CBM is rapidly metabolized in the blood to MMI and is on average 2 times less potent than MMI at the same dose (Jansson et al., 1983). Propyl thiouracil (PTU) is the least potent compound at the same dose (10 times less potent than MMI). MMI is considered as the standard ATD with the highest efficacy due to its acceptable and low side effects as well as longest half-life (Cooper, 2003; 2005). However, the efficacy of ATD treatment is limited. Patients with persistent TRAB elevation or hyperthyroidism at 18th month of maintenance therapy, or patients with recurrence after completing the MMI course, can also choose radical treatment with RAI or total thyroidectomy (TX). TX should be performed by an expert who performs considerable thyroid surgeons, while RAI should be avoided in GD patients with active GO or with a history of smoking (Kahaly, 2020).

However, there are many negative effects of currently available GD treatments. In a study, 2,430 newly diagnosed GD patients were recruited from 13 Endocrine clinics in Sweden, and it found remission rates were 45.3% (351/774) for first-line treatment with ATD, 81.5% (324/264) for I131, and 96.3% (52/54) for surgery (Starling, 2019). The remission rate was even lower (29.4%) if a second round of ATD was given to patients who had relapsed after receiving ATD. Frequent relapses are the main problems and large goiter size was significantly associated with an increased recurrence hazard ratio. Patients who choose ATD as first-line treatment should be informed that they have only a 50.3% chance of avoiding ablation and only a 40% chance of long-term normal thyroid function (Starling, 2019). To help the clinician to tailor a treatment for newly diagnosed Graves’ hyperthyroidism in real life, the GREAT (Graves’ Recurrent Events After Therapy) score and the Clinical Severity Score (CSS) have been developed, which are useful tools to predict at baseline relapse of hyperthyroidism after treatment (Masiello et al., 2018).

The risk of hypothyroidism with levothyroxine (LT) 4 therapy after radioiodine (RAI) treatment is significantly higher than that during long-term ATD therapy. What’s more, RAI therapy has been significantly associated with GO exacerbation, partly due to increased TRAB titers after RAI. In the most robust study, 443 GD patients were randomized to receive RAI or methimazole. The frequency of GO occurrence or progression was significantly higher in the RAI group (15%) than those in the MMI group (2%) (OR 6.5 [95% CI: 2.2 -- 19.4]) (El Kawkgi et al., 2021). However, oral steroid prophylaxis for Graves’ orbitopathy after radioactive iodine treatment for Graves’ disease is not only effective, but also safe (Rosetti et al., 2020).

As for thyroid surgery, patients need life-long thyroxine replacement therapy and possibly have abnormal low circulating parathyroid hormone (PTH) levels after surgery, which can lead to disorders of the calcium-phosphate balance (Jørgensen et al., 2021). A study including 7,366 thyroidectomy patients showed that patients with severe hypocalcemia had a higher rate of recurrent laryngeal nerve injury (13.4% vs. 6.6%), unplanned reoperation (4.4% vs. 1.3%), and longer hospitalization (30.4% vs. 6.2%) (*p* < 0.01) (Kazaure et al., 2021).

## New Treatment Strategies for Graves’ Hyperthyroidism

The need for new treatment regimens, combined with a better understanding of basic immunobiology, has led to the emergence of new approaches to treat graves’ hyperthyroidism. Therapies currently under investigation include biologics, small molecule peptides, immunomodulators and teprotumumab (Kahaly, 2020). Additionally, Th1 immune response prevails in the immune-pathogenesis of GD and GO, during the active phase, when Th1 chemokines, and their (C-X-C) R3 receptor, play a key role. In thyrocytes, the inhibition of Th1 chemokines secretion was stronger with peroxisome proliferators-activated receptors (PPAR)-α than PPAR-γ ligands (90% with fenofibrate and 85% with ciprofibrate), suggesting that PPAR-*α* can modulate the immune response (Antonelli et al., 2020).

### Depletion of B Lymphocytes (CD20 Depletion)

As a B-cell depletion therapy, Rituximab (RTX) has been used to treat lymphoproliferative malignancies such as lymphoma for more than 20 years and has been increasingly used in autoimmune diseases over the past decades. Although anti-CD20 monoclonal antibody RTX is widely studied of B cell therapies, the exact mechanism by which RTX has beneficial effects remains uncertain. (Pavanello et al., 2017).

### Disruption of B Cell Activation or Activity (Blocking CD40 Interactions)

Anti-CD40 monoclonal antibody Iscalimab (CFZ533) targets the CD40-CD154 costimulatory pathway, resulting in reduced B cell activation signaling (Ristov et al., 2018). Iscalimab is a non-consumable immunoglobulin silencing antibody designed to block CD40 receptor interactions without removing CD40-expressing cells. Like RTX, Iscalimab is another immunosuppressive therapy (Pavanello et al., 2017).

### Blocking Immunoglobulin Recirculation (FcRn Therapy)

Neonatal immunoglobulin receptor (FcRn) is associated with the long half-life of IgG antibodies such as TRAB. FcRn participates in the recycling process by binding to IgG antibodies through endocytosis under acidic conditions in the lysosome, after which a number of IgG copies are released back into the tissue to participate in the immune response (Smith et al., 2018). Inhibition of FcRn is an attractive new therapeutic concept, in which accelerated antibody catabolism and reduced circulating pathogenic TRAB levels are beneficial for GD treatment. The two most widely studied compounds targeting FcRn are efgartigimod and Rozanolixizumb (Zuercher et al., 2019), both of which are currently in phase 3 trial to treat autoimmune diseases. Efgartigimod is a humanized IgG-1 derived Fc fragment, while Rozanolixizumab is a humanized anti-FcRn monoclonal antibody, both of which can block FcRn-IgG interaction. (Kiessling et al., 2017; Smith et al., 2018).

### Inhibiting B Cell Proliferation and Differentiation (Blocking BAFF)

BAFF is a member of the TNF family of cytokines that play an important role in B lymphocyte activation, differentiation, and survival. The increased expression of BAFF and its major receptor (BAFF-R) in infiltrating immune cells and thyroid cells of GD patients suggests that BAFF-BAFF-R interaction plays a key role in the pathogenesis of GD. (Lin et al., 2016). BAFF monoclonal antibody belimumab binds to and antagonizes the bioactivity of soluble BAFF. Blocking the interaction between BAFF and its receptor has a negative effect on B cell proliferation, indirectly reducing B cell survival rate and reducing the production of autoantibodies (Stohl et al., 2012; Campi et al., 2015).

### Specifically Targeting TSHR

Including small molecule TSHR antagonist Antag-3, VA-K-14, S37a, K1-70, etc (Neumann et al., 2010; Neumann et al., 2014). Antag-3 inhibits TSH-stimulated cyclic adenosine phosphate (cAMP) production *in vitro* and reduces thyroid hormone levels in mice treated with thyroid-stimulated monoclonal antibody M22. Two other TSHR antagonist compounds, VA-K-14 and S37a, have been identified by high-throughput library screening. They can both inhibit TSH expression and TRAB-induced signaling *in vitro* (Latif et al., 2016; Marcinkowski et al., 2019). TSHR-blocking antibody K1-70, which could completely inhibit the elevation of serum thyroxine, suggesting a potential therapeutic effect for GD with high serum TRAB level (Furmaniak et al., 2012). Specific immunotherapy against TSHR involves the use of drugs with a broad immunosuppressive effect and therefore has the potential for infectious side effects.

### IGF-1R Inhibitor

There is considerable evidence show that IGF-1R is meaningfully involved in the development of GO (Smith and Janssen, 2019). Orbital fibroblasts, T cells and B cells overexpress IGF-1R (Pritchard et al., 2003; Douglas et al., 2007; Douglas et al., 2008), there is a functional collaboration between IGF-1R and TSHR (Tsui et al., 2008), while TSHR in GD patients targeted by TRAB causes pathological symptoms (Pritchard et al., 2002; Pritchard et al., 2003). Teprotumumab, an IGF-1R inhibitor, is the only Food and Drug Administration (FDA)-approved treatment for GO based on the understanding that IGF-1R plays an important role in the pathogenesis of GO (Markham, 2020).

Newly developed biological agents, small molecules, and peptide treatment such as immune regulator also have potential limitations due to the unclear beneficial effects and exact mechanism. Besides, it is unknown whether these agents will improve the long-term risk of hypothyroidism, decrease overactive goiter and prevent the late recurrence. Combined with the high cost and potential risk of immune damage from nonspecific treatments, such as infusion reactions, gastrointestinal symptoms, and severe infections, common use of these drugs in adults with GD is not currently recommended (Lane et al., 2020).

## Effective Ingredients From Traditional Chinese Medicine and Action Mechanisms for GD

Ingredients of traditional Chinese medicine are attracting increasing interests in modern medicine, as some ingredients have been successfully used to treat autoimmune diseases (Ma et al., 2013; Ma and Jiang, 2016; Shen and Wang, 2018).

### Diosgenin From *Trigonella foenum-graecum* L [Fabaceae; Common Fenugreek Seed] or *Dioscorea Bulbifera* L [Dioscoreaceae; Sevenlobed Yam Rhizome]

**FIGURE 2:**
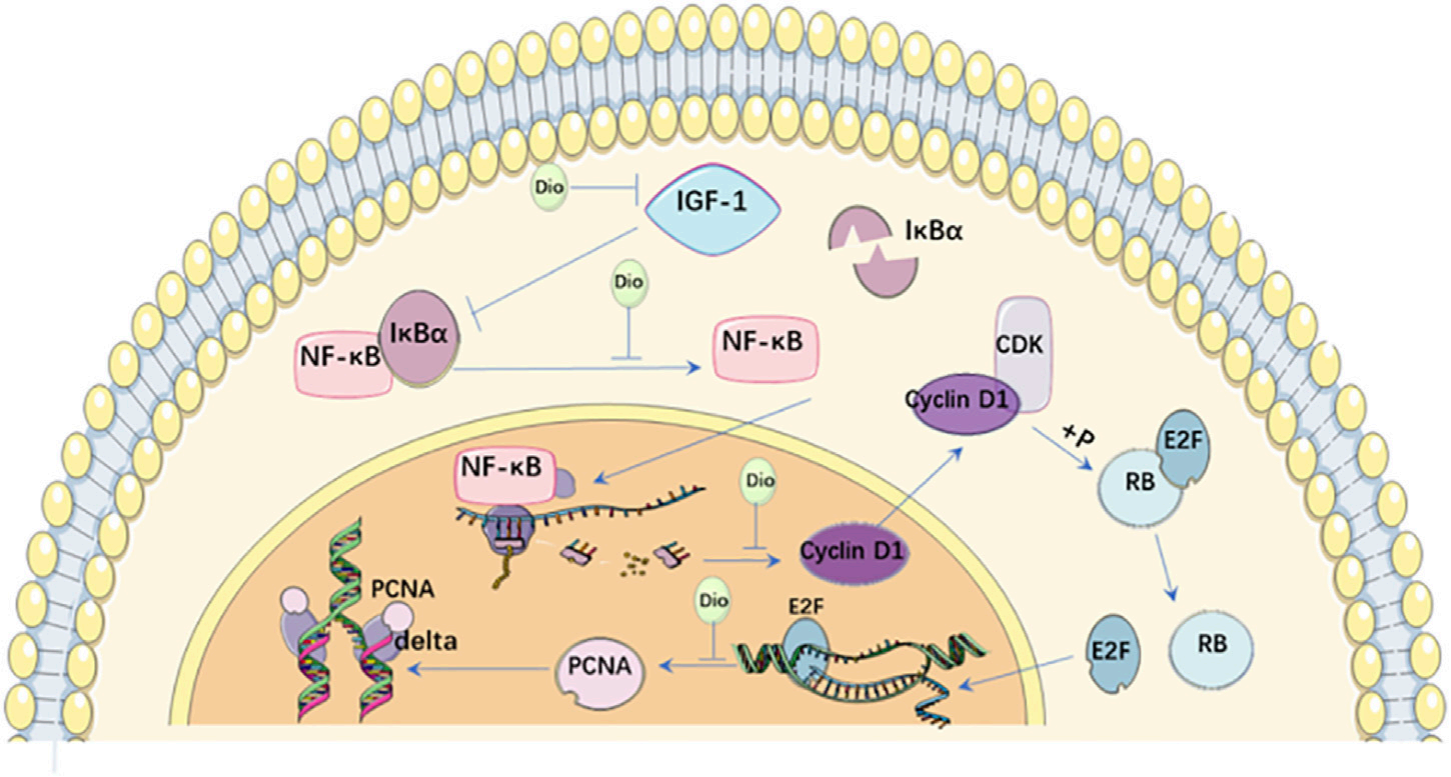
Action mechanisms of Dio on Graves’ disease.

Diosgenin (Dio) is a natural steroid saponin, which is produced in large quantities in *Trigonella foenum-graecum* L [Fabaceae; Common fenugreek seed] and *Dioscorea bulbifera* L [Dioscoreaceae; Sevenlobed yam rhizome ] (He et al., 2012). Dio had control effects on goiter and hyperthyroidism in GD mice. Interestingly, thyroid hormone tatalthyroxine (TT4) expression and thyroid size in normal mice were only slightly affected, and the difference was not statistically significant even after high-dose Dio treatment. Thus, Dio selectively affects the proliferating thyroid rather than the normal thyroid, suggesting that Dio may be a safe anti-goiter agent to avoid hypothyroidism. The target of Dio may not be expression of TRAB, but thyroid cell proliferation (Cai et al., 2014). In addition to TRAB, several other growth factors are involved in the proliferation of GD cells, among which IGF-1 is considered as the most important factor (Völzke et al., 2007). Dio inhibits IGF-1-induced thyroid cell proliferation *in vitro* by decreasing the expression of IGF-1, nuclear factor-k-gene binding (NF-κB), cyclin D1 and proliferating cell nuclear antigen (PCNA). In mouse thyroid culture cells (FRTL), IGF-1 promotes cell cycle progression by up-regulating G1/S-specific cyclin D1 through activation of NF-κBpathway (Ren et al., 2009). NF-κB is a protein complex that plays a key role in regulating cell proliferation and cell survival. In the inactive state, NF-κB is located in the cytoplasm and binds to inhibitory protein recombinant inhibitory subunit of NF kappa B alpha (IκBα). As a stimulus, IGF-1 induces phosphorylation and degradation of IκBα, leading to NF-κB activation. Activated NF-κB enters the nucleus and activates transcription of target genes (Salminen and Kaarniranta, 2010). Cyclin D1 co-phosphorylates retinoblastoma (RB) protein with cyclin-dependent kinase (CDK) and promotes the release of binding E2F transcription factor. These events promote transcription of E2F target genes and participate in the entry and completion of S phase (Stacey, 2003). One E2F target gene is PCNA, a cofactor of the DNA polymerase delta, which is required for DNA synthesis in the S phase (Travali et al., 1989). This study demonstrated that Dio could simultaneously inhibit the overexpression of these proliferation-related proteins in the thyroid of GD mice, suggesting that Dio is a potential new drug candidate for the treatment of GD. Because Dio has multiple biological activities, this compound may affect multiple molecules of the proliferation pathway or pathological aspects of GD (Cai et al., 2014). (Figure 2)

IGF-1 induces phosphorylation and degradation of IκBα, leading to NF-κB activation. Activated NF-κB enters the nucleus and activates transcription of Cyclin D1. Cyclin D1 co-phosphorylates RB protein with CDK and promotes the release of binding E2F transcription factor from RB. Then E2F targets at the expression of gene PCNA, a cofactor of the DNA polymerase delta, and promotes the transcription and translation of downstream proliferation-related proteins. Dio can decrease the expression of IGF-1, NF-κB, Cyclin D1 and PCNA to inhibit thyroid cell proliferation.

Dio, Diosgenin; IGF-1, Insulin-like growth factor-I receptor; NF-κB, nuclear factor-k-gene binding IκBα, recombinant inhibitory subunit of NF kappa B alpha; PCNA, proliferating cell nuclear antigen; CDK, cyclin-dependent kinase; E2F, E2F transcription factor; RB, retinoblastoma.

### Resveratrol From *Reynoutria japonica* Houtt [Polygonaceae; Polygoni Cuspidati Rhizoma et Radix]

Resveratrol is the active ingredient in *Reynoutria japonica* Houtt [Polygonaceae; Polygoni cuspidati rhizoma et radix] (Ravagnan et al., 2013). Resveratrol is a stilbenoid produced by plants in response to injury and is associated with increased levels of Cu/Zn superoxide dismutase and glyoxal oxidase (Lucini et al., 2018). Resveratrol was found to reduce oxidative stress in GO patients, and increased the nuclear and transcriptional activity of Nuclear Factor 2 (NRF2), thereby increasing its ability to bind to antioxidant genes (ARE) (Gong et al., 2017). NRF2 is an zipper protein with alkaline leucine. Under normal conditions, it is retained in the cytoplasm by Kelch-like ECh-associated protein 1 (Keap1) and degraded by specific cyclin 3 (CUL3) (Itoh et al., 1999). Oxidative stress disrupts the KEAP1-CUL3 ubiquitination system by interacting with the cysteine residues of Keap1, thereby allowing NRF2 to be transported into the nucleus, binding to ARE and promoting downstream product expressions (Dinkova-Kostova et al., 2002; Yamamoto et al., 2008). Some studies have shown that oxidative stress is related to the pathogenesis of GO. In addition, some studies have shown that GO orbital fibroblasts are hypersensitive to oxidative stress. Resveratrol can reduce the production of reactive oxygen species (ROS) and human heme oxygenase 1 (HO-1) induced by oxidative stress, and inhibit lipogenesis and lipid droplet accumulation (Kim et al., 2015). Resveratrol enhances the nuclear translocation of NRF2 in cultured orbital fibroblasts, promotes the activation of NRF2-ARE pathway, and induces the expression of antioxidant gene ARE. However, NRF2 silence decreases the protective effect induced by Resveratrol in orbital fibroblasts. In conclusion, Resveratrol can relieve oxidative stress-related symptoms by stimulating the NRF2-ARE pathway and inhibit adipogenesis of orbital fibroblasts *in vivo* by reducing ROS production (Li et al., 2020).

### Icariin From *Epimedium Brevicornu* Maxim [Berberidaceae; Epimedium Alpinum Aboveground Part or Leaf]

Icariin inhibits the differentiation of preadipocytes into mature adipocytes by inhibiting autophagy, and these effects are mediated by inhibiting the activation of the 5 ′-adenosine phosphate activated protein kinase/mechanistic target of rapamycin (AMPK/mTOR) pathway (Li et al., 2017). In a thyrotropin receptor-induced GO mouse model, Icariin reduces adipose tissue dilation in orbital muscle and lipid drop accumulation by inhibiting AMPK/mTOR mediated autophagy (Li et al., 2017). In addition, a decoction including *Epimedium brevicornu* Maxim [Berberidaceae; Epimedium alpinum aboveground part or leaf], Pingmu decoction, can reduce the accumulation of orbital adipose cells in GO inactive phase to play a therapeutic role, which may be explained by increased expressions of death receptor (Fas)/death receptor ligand (Fas L) and apoptosis. Pingmu decoction can reduce the cell viability of preorbital adipocytes, inhibit their adipocyte differentiation, and promote the apoptosis of mature adipocytes by activating death signaling pathways through Fas and Fas L. These results suggested the therapeutic mechanism of Pingmu decoction in reducing the accumulation of orbital adipose cells in GO development (Zhang et al., 2017).

### Celastrol From *Celastrus orbiculatus* Thunb [Celastraceae; Celastrus Orbiculatus Stem]

Celastrol, a triterpenoid compound isolated from Traditional Chinese medicine like *Celastrus orbiculatus* Thunb [Celastraceae; Celastrus orbiculatus stem], is a promising drug for the treatment of various inflammatory and autoimmune diseases. Cytokines play a key role in the development of GO and are essential for the development and maintenance of inflammation. It has been reported that Interleukin (IL)-1β mRNA expression level is higher in orbital tissues of GO patients, and IL-1β mediates inflammatory response (Wakelkamp et al., 2003). Celastrol significantly inhibited IL-1β, and thus inhibited IL-1β-induced production of orbital fibroblast cytokines IL-6, IL-8, intercellular adhesion molecule (ICAM-1), and cyclooxygenase-2(COX-2) (Chen and Greene, 2004; Konuk et al., 2006). ICAM-1 expression is involved in the migration of lymphocytes to orbital inflammatory sites (Sikorski et al., 1993). COX-2 is also considered to be the key to the inflammatory response of GO patients, and the expression of COX-2 is positively correlated with the increasing severity of orbital diseases (Konuk et al., 2006). IL-1β promotes orbital fibroblasts in patients with GO producing high levels of COX-2 through activation of the NF-κB pathway. Celastrol inhibits IL-6, IL-8, ICAM-1, and COX-2 cytokines production in IL-1β-induced orbital fibroblasts, which suppresses inflammation and inhibits the progression of GO (Li et al., 2016).

### Gypenosides From *Gynostemma pentaphyllum* (Thunb.) Makino [Cucurbitaceae; Gynostemma PentaphyllumAerial Part]

Gypenosides are saponins extracted from *Gynostemma pentaphyllum* (Thunb.) Makino [Cucurbitaceae; Gynostemma pentaphyllumaerial part ]. They are the pharmacological active components in gynost’ pentaphyllum and have a variety of biological activities. Gypenosides can regulate the activation of immune cells and the expression of cytokines, and inhibit the inflammatory response of different diseases (Wang et al., 2017; Wang et al., 2018). Gypenosides have anti-inflammatory and antioxidant biological effects. Through GO analysis, PPI network construction and molecular docking, it is found that Gypenosides may play anti-inflammatory and antioxidant roles in GO through signal transducer and activator of transcription (STAT1) and STAT3 signaling pathways. Inhibiting the expression of STAT1 signaling pathway, Interferon (IFN)-γ-induced productions of chemokine 10 (IP-10)/CXC-chemokine ligand 10 (CXCL10) in orbital fibroblasts of GO patients can be inhibited, thus alleviating orbital inflammation (Pu et al., 2019). The STAT3 signaling pathway plays an anti-inflammatory, antioxidant and immunomodulatory role in chronic respiratory diseases, breast cancer and liver inflammation (Alhusaini et al., 2018; Natarajan et al., 2019; Xiang et al., 2019). Inflammation and oxidative stress damage of orbital tissues are the main pathogenesis of GO (Li et al., 2016; Rotondo Dottore et al., 2017). Therefore, STAT1 and STAT3 signaling pathways may be the key target signaling pathways for Gynostevenosides in the treatment of GO, reducing tissue damage and remodeling caused by orbital inflammation and oxidation (Li et al., 2019).

### Astragaloside IV From *Astragalus mongholicus* Bunge [Fabaceae; Astrgali Mongholici Radix]

Astragaloside IV (AS-VI) is the main active ingredient of *Astragalus mongholicus* Bunge [Fabaceae; Astrgali mongholici radix]. AS-VI has antioxidant and anti-inflammatory properties and has shown therapeutic potential in numerous ischemic and inflammatory diseases (Li et al., 2018). IL-1β increases the mRNA expression of inflammatory cytokines IL-6, IL-8, TNF-α and monocyte chemotactic protein-1 (MCP-1) in cultured orbital fibroblasts. This IL-1β -induced inflammation is accompanied by increased autophagy activity, reflected in increased expression of the autophagy effector proteins Beclin-1 and angiotensinogen (AGT)-5 and the conversion of autophagy markers microtubule-associated protein light chain 3 (LC3)-I to LC3-II. Preconditioning with the autophagy inhibitors 3-methyladenine (3-MA) and Bafilomycin A1, or silencing the autophagy related proteins Beclin-1 and ATG-5, could prevent IL-1β-induced orbital fibroblast inflammation, while preconditioning with the autophagy activator rapamycin had the opposite effect. These data suggests that autophagy is involved in GO orbital inflammation. AS-VI treatment significantly reduced IL-1β-induced inflammatory cytokine production *in vitro* and reduced GO orbital inflammation, fat accumulation, collagen deposition, and macrophage infiltration *in vivo*. The protective effect of AS-IV on GO was also associated with decreased autophagy activity of orbital fibroblasts and orbital tissues respectively (Li et al., 2018).

### Ingredients From *Prunella vulgaris* L [Lamiaceae; Prunellae Spica Fruit]

Spica Prunellae (SP), the fruit of *Prunella vulgaris* L [Lamiaceae; Prunellae spica fruit], is a traditional antipyretic botanical drug widely distributed in Northeast Asia (Zhang et al., 2020). SP has been widely used in thyroid diseases, such as goiter and subacute thyroiditis (Li et al., 2019). And in many herbal formulations, SP is treated as an important ingredient for the treatment of GO (Yang et al., 2007). According to the compound-hub gene-pathway network, Quercetin, Ursolic acid and Rutin interacted with the large number of targets, indicating the main active ingredients in SP and the important roles in the anti-GO system (Zhang et al., 2020). Quercetin is a flavonoid phytoestrogen exhibiting antioxidant and anti-inflammatory properties and reducing proliferation in orbital fibroblasts. Ursolic acid and Rutin are reported to promote apoptosis and regulate immune systems in cell and animal models (Zhang et al., 2020). The PI3K-Akt signaling pathway plays a key role in both immune inflammation and proliferation and apoptosis in GO, and this process may be an effective therapeutic target for SP (Bahn, 2010). In terms of immune inflammation, pro-inflammatory cytokines COX-2, IL6 and TNFα are confirmed to be involved in the pathogenesis of GO. Previous studies have shown that COX-2 decreased with declined GO clinical activity scores and is now considered to be critical to the inflammatory process in patients with GO (Dubois et al., 1998; Konuk et al., 2006; Vondrichova et al., 2007). COX-2 is involved in the biosynthesis of prostaglandins, which plays a key role in inflammation. IL6 is related to the pathogenesis of autoimmune diseases and the AKT/NF-κB signaling pathway has been reported to contribute to IL6 production in the retrobulbar space during GO activity (Gillespie et al., 2012). Similarly, elevated serum TNF levels in GO inflammation have been shown to be mediated by the AKT/NF-κB signaling pathway. Currently, some TNF inhibitors, like SP, have been reported to achieve promising results in patients with GO, regardless of rare adverse reactions (Kapadia and Rubin, 2006). For proliferation and apoptosis (Kumar et al., 2011; Li and Smith, 2014; Woeller et al., 2019), the PI3K-Akt signaling pathway seems to play an important role in mediating cell growth and death in GO. Recent studies have shown that orbital fibroblasts overexpress TSHR and increase the expression and proliferation of inflammatory genes by activating the PI3K-Akt pathway. In addition, the PI3K-Akt signaling pathway is also involved in cell proliferation of preorbital adipose cells (Wang et al., 2019), thus promoting GO progression. Caspase 3 (CASP3) activation is one of the last steps of apoptosis, and SP shows a pro-apoptotic effect in GO by activating CASP3 (Zhu et al., 2018).

### Triptolide From *Tripterygium wilfordii* Hook.f [Celastraceae; Tripterygium Wilfordii Radix]

Triptolide, a diterpenoid tricyclic oxide compound purified from the roots of *Tripterygium wilfordii* Hook. f [Celastraceae; Tripterygium wilfordii radix], has been identified as one of the main components responsible for the immunosuppressive properties of this botanical drug (Gu et al., 1995). The immunosuppressive activity of Triptolide has been studied *in vitro* and *in vivo*, and it has been found to inhibit T cell proliferation, induce T cell apoptosis, reduce IL-2 synthesis, and inhibit the expression of NF -κB in T cells (Yang et al., 1994; Yang et al., 1998; Li et al., 2002; Qiu and Kao, 2003). Triptplide could relieve the clinical manifestations of exophthalmos, diplopia and periorbital swelling caused by inflammatory cell infiltration and accumulation of adipose tissue in the extraocular muscle and orbital connective tissue. Abnormal expression of human leukocyte antigen -DR (HLA-DR) on fibroblasts is associated with the development of GO (Heufelder et al., 1991; Hiromatsu et al., 1995), and it has been reported that the expression of various adhesion molecules (e.g., ICAM-1) on orbital fibroblasts (RFs) is involved in the migration of lymphocytes to the site of orbital inflammation. The cell surface molecule CD40 is a key signal molecule for B lymphocyte expression, and it has been established that CD40 is expressed in RFs and plays an important role in the interaction between RFs and T lymphocytes. The expression of HLA-DR, ICAM-1, and CD40 are all induced by IFN-γ. Triptolide inhibited IFN-γ-induced RFs activation in GO patients, decreased the expression of HLA-DR, ICAM-1 and CD40, and inhibited cell proliferation and hyaluronic acid (HA) synthesis in GO patients (Yan and Wang, 2006).

### Bupleurum Saponins From *Bupleurum falcatum* L [Apiaceae; Bupleuri Radix]

Bupleurum saponins, the active component of *Bupleurum falcatum* L [Apiaceae; Bupleuri radix], which have strong antioxidant effects, can improve hyperthyroidism and related organ damages induced by Levothyroxine (LT4). They have good bidirectional regulation effects on hyperthyroidism and secondary hypothyroidism (Kim et al., 2012). In addition, some traditional Chinese medicines with good efficacy are widely used in clinical practice, such as *Prunella vulgaris* L [Lamiaceae; Prunellae spica fruit], *Bupleurum falcatum* L [Apiaceae; Bupleuri radix], *Fritillaria thunbergii* Miq [Liliaceae; Fritillaria thunbergii bulb] and *Paeonia lactiflora* Pall [Paeoniaceae; Paeonia species flower et root], etc. The active components and mechanisms are to be thoroughly studied.

## Prospects of Traditional Chinese Medicine on Treating GD

### Traditional Chinese Medicine Relieves Large Goiter and Reduce Side Effects of ATD

Many botanical drugs or ingredients could reduce large goiter. For example, Dio reduced goiter formation in GD patients, and the effect was independent of TRAB levels, and the underlying mechanism involved inhibiting thyroid cell proliferation by inhibiting gene transcription and protein expression of certain proliferation-related proteins. Some Chinese botanical drugs can induce apoptosis in combination with ATD in GD. A study including 13 patients of Graves’disease showed that compared with ATD alone, thyroid volume decreased significantly after combined treatment (*p* < 0.01) (Zhao et al., 1999). Typical apoptotic appearances, such as vacuolar cells, marginal nuclei, chromatin aggregation, and nuclear fragmentation, could be seen under light microscopy, and the apoptosis rates are 2.11 % and 18.66% before and after administration (*p* < 0.01) (Zhao et al., 1999). Moreover, Dio selectively affects the proliferating thyroid rather than the normal thyroid, suggesting that Dio may be a safe anti-goiter agent to avoid hypothyroidism (Cai et al., 2014). This suggests that traditional Chinese medicine can make up for the deficiency in ATD therapy and effectively relieve the enlargement of abnormal thyroid volume without causing secondary hypothyroidism or other adverse reactions.

### Traditional Chinese Medicine Relieves Symptoms of GO

In addition to the inhibition of orbital fibroblasts to release hydrophilic polysaccharide and proinflammatory cytokines (Armengol et al., 2001; 2020) to alleviate the orbital inflammatory process and local symptoms such as edema, hyperemia and exophthalmos, some effective components of traditional Chinese are effective in reducing the accumulation of orbital fat cells, reducing orbital pressure and improving malignant exophthalmus. For example, resveratrol can enhance oxidative stress and inhibit the adipogenesis and accumulation of lipid droplets, Icariin inhibits the differentiation of preadipocytes into mature adipocytes by inhibiting autophagy; Epimedium glycoside can reduce the vitality of preorbital fat cells, restrain adipocyte’s differentiation, and activate death signaling pathways through Fas and Fas L to promote the apoptosis of mature fat cells; and SP promotes adipocyte apoptosis by activating CASP3 in GO.

### Traditional Chinese Medicine Alleviates the Hypermetabolic Symptoms of GD

As for the relief of symptoms in patients with hyperthyroidism, Astragalus can regulate the immune function of GD patients and significantly reduce the levels of serum IL-β, TNFα, IL-6, IL-8 and MCP-1, thus relieving hyperhidrosis, palpitation and other clinical symptoms, which plays an important role in the adjuvant treatment of GD (Wu et al., 2011). *Dendrobium officinale* Kimura and Migo [Orchidaceae; Dendrobium stem] (DOF) can reduce the liver function injury caused by overactive thyroid axis by affecting thyroxine metabolism, reduce blood flow in microcirculation of face and ears, lower the facial temperature and heart rate, and alleviate symptoms such as zygotic redness, irritability and liver function damage in patients (Lei et al., 2015).

### Traditional Chinese Medicine Reduces Allergic Symptoms and Increases the Dosage of ATD Used in Allergic Patients

When patients with hyperthyroidism take antithyroid drugs, drug allergy is easy to occur. The main manifestations are skin fever, itching, maculopapule or urticaria on the face and trunk. In addition to alleviating the symptoms of hyperthyroidism, some Chinese medicines can also alleviate ATD allergic reaction. The compound hyperthyroidism tablet, which contains nine kinds of botanical drugs directed at GD and small dose MMI, has a good effect on the hyperthyroidism with deficiency of qi and Yin, and can be effective in desensitization and treatment of drug rash (Wang, 2000). For patients who refuse surgery or RAI treatment, gradually increasing the treatment dose of ATD assisted by Traditional Chinese medicine may be a treatment choice for them. However, the number of relevant researches is small, and more efforts are still needed to conduct drug screening and mechanism research.

### Limitations and Research Direction of Traditional Chinese Medicine in the Treatment of GD

Although some botanical drugs and effective components can compensate for the deficiency of existing treatments, the researches of Chinese medicine are still limited in quantity and quality. The existing studies were inclined to improve the exophthalmos of GD and the goiter, relevant symptoms of nervous, cardiovascular, digestive, reproductive, skin and skeletal systems caused by hyperthyroidism were ignored. What’s more, thyroid function - with the exception of restored thyroid stimulating hormone (TSH) - was not significantly altered by TCM (Zen et al., 2007). A systematic review included in 17 randomized controlled clinical trials with 1,536 participants showed that the serum glutamic pyruvate transaminase (SGPT) of participants slightly increased and menstrual quantity decreased during TCM treatment, but all of the aforementioned studies indicate that the occurrence rates of reported adverse effects in TCM intervention groups were fewer than controls (Xu et al., 2014). Three studies reported drug-induced symptoms (such as nausea, vomiting, and gain weight), and the respondents had fewer adverse symptoms in the TCM intervention groups compared with the controls (RR: 0.32; 95% CI: 0.20–0.53; *p* < 0.00001; fixed model; I2 = 0%; three trials; n = 197) (Liu et al., 2019). What’s more, majority of them were mild and tolerable and disappeared spontaneously after reducing the dosage of TCM or drug withdrawal. Four studies did not provided clearly proportions, and other studies still at the stage of compound preparation or clinical efficacy evaluation and did not report the safety events. Some traditional Chinese medicines are widely used in clinical practice and have good efficacy, such as *Prunella vulgaris* L [Lamiaceae; Prunellae spica fruit], *Bupleurum falcatum* L [Apiaceae; Bupleuri radix], *Fritillaria thunbergii* Miq [Liliaceae; Fritillaria thunbergii bulb] and *Paeonia lactiflora* Pall [Paeoniaceae; Paeonia species flower et root]; however, relevant studies to explore the effective molecules of traditional Chinese medicine and their exact mechanisms were seriously deficient, which also points out the direction we need to work on in the future.

The current researches about GD with single drug are numerous, such as TCM containing high-level iodine (*Laminaria japonica* Aresch [Laminariaceae; Ecklonia kurome leaf], *Dioscorea bulbifera* L [Dioscoreaceae; Aerial yam aerial parts et rhizome], FossiliaOssisMastrodi, Ostrea gigas tnunb, *Prunella vulgaris* L [Lamiaceae; Prunellae spica fruit], etc.), botanical drugs with immunosuppressive effects (*Tripterygium wilfordii* Hook. f [Celastraceae; Tripterygium wilfordii radix], *Dioscorea bulbifera* L [Dioscoreaceae; Sevenlobed yam rhizome], *Malus toringo* (Siebold) de Vriese [Rosaceae; Malus spectabilis flower], *Ranunculus ternatus* Thunb [Ranunculaceae; Ranunculi ternati radix], etc.), botanical drugs with immunomodulatory effects (*Astragalus mongholicus* Bunge [Fabaceae; Astrgali mongholici radix], *Scrophularia ningpoensis* Hemsl [Scrophulariaceae; Scrophulariae radix], *Paeonia lactiflora* Pall [Paeoniaceae; Paeonia species flower et root], *Anemarrhena asphodeloides* Bunge [Asparagaceae; Anemarrhenae rhizoma], *Rehmannia glutinosa* (Gaertn.) DC [Orobanchaceae; Rehmanniae radix], Carapax Trionycis, *Dendrobium officinale* Kimura and Migo [Orchidaceae; Dendrobium stem], etc.) and other traditional Chinese medicines with good clinical effect (*Bupleurum falcatum* L [Apiaceae; Bupleuri radix], *Gentiana scabra* Bunge [Gentianaceae; Gentianae radix et rhizoma], *Prunella vulgaris* L [Lamiaceae; Prunellae spica fruit], *Calamus draco* Willd [Arecaceae; Dragon’s blood palm], etc.).

The composition of Chinese traditional medicine is complex and the molecular targets are multiple, which are the main causes of its unsure clinical efficacy. But with the development of biochemistry, molecular biology, immunology, pharmacology, chemistry and pharmacology of plants, and the emergence of new methods and technologies such as surface plasmon resonance (SPR) analysis, drug affinity reaction target stability (DARTS) analysis, Chinese medicine component chip, drug molecular target hook, screening analysis platform and pathway enrichment analysis, the relevant scientific research work has been continuously improved, and the mechanism exploration of effective molecules and exact targets of single Chinese medicine have gradually become clear. In general, we should still focus on efficacy and screening out the ingredients of TCM with better efficacy and clear mechanism. There is still a long way to go to analyze and extract effective molecules of botanical drugs, prepare finished drug product and promote the modernization, quality and standardization, and finally promote the high-level clinical service with new methods and technologies.

## Conclusion

The inadequacy of traditional treatment promotes the emergence of new therapeutic ideas, including biologics, small molecule peptides, immunomodulators and specific antibody IGF-1R. However, due to the precise mechanism of the treatment effect is unknown and the risk of complications during treatment process, they have not been actively put into clinical use. What’s more, these new treatments are mostly targeting the upstream and downstream of the abnormal activation of TSHR to reduce the abnormal thyroid hormone secretion, and lacking relevant research and treatment strategies for abnormal hyperplasia and hypertrophy of thyroid follicular epithelial cells, accumulation of orbital adipose cells and formation of lipid drops in patients with GO, hyper sweating, palpitations, zygomatic redness, irritability, impaired liver function and anaphylactic reaction towards ATD. Some active ingredients can inhibit the excessive proliferation of thyroid follicular epithelial cells and relieve eye symptom and systemic manifestation of GD, as well as playing roles in desensitization and treatment of ATD related allergic reaction. But due to the exility of number and the lack of depth, many studies only stay in the clinical efficacy evaluation stage of compound preparations, and lacking specific verification of effective molecules and molecular mechanisms of actions in single element. Though some adverse effects of TCM had been reported, such as menstrual disorders, gastrointestinal events, impaired liver function and rash, they were mild and recovered after the decrease of TCM dose. The effective components of Traditional Chinese medicine might open a new window for the treatment of GD, but high-quality RCT studies and the exact mechanisms still need to be further explored.
